# Dual-target immunotherapies in NSCLC: a systematic review and meta-analysis of randomized clinical trials

**DOI:** 10.3389/fimmu.2025.1605877

**Published:** 2025-09-10

**Authors:** Yike Zhang, Haozhe Wang, Xinyue Yang, Changhai Lei

**Affiliations:** Department of Biophysics, College of Basic Medical Sciences, Naval Medical University, Shanghai, China

**Keywords:** bispecific antibodies, NSCLC, immunotherapy, meta-analysis, dual-target immunotherapies

## Abstract

**Background:**

Despite advances in targeted therapies and immune checkpoint inhibitors (ICIs), the prognosis for advanced non-small cell lung cancer (NSCLC) remains poor. Bispecific antibodies (BsAbs) represent an emerging class of dual-target immunotherapies, yet their comparative efficacy and safety profiles lack comprehensive quantitative synthesis.

**Methods:**

This systematic review and meta-analysis (PROSPERO CRD420251005168) adhered to PRISMA guidelines. We systematically searched PubMed, Web of Science, Scopus, and Embase through March 2025 for phase III randomized controlled trials (RCTs) comparing dual-target immunotherapies with conventional therapies in advanced NSCLC. Primary outcomes were progression-free survival (PFS) and overall survival (OS); secondary outcomes included objective response rate (ORR), disease control rate (DCR), and treatment-related adverse events (AEs). Risk of bias was assessed using Cochrane RoB 2.0. Random-effects models were used for data synthesis.

**Results:**

Six RCTs (*n*=3,063 patients) were included. Dual-target immunotherapies significantly improved PFS (HR= 0.58, 95% CI: 0.43-0.78; *p*<0.001) and ORR (RR=1.29,95%CI: 1.01-1.64; *p*=0.04) compared to conventional therapies. No significant OS (HR=0.84,95% CI: 0.68-1.05; *p*=0.13) or DCR (RR=1.09, 95% CI: 0.92-1.30; *p*=0.30) benefits were observed. Subgroup analyses stratified by mechanism showed no statistically significant differences in efficacy and safety between dual-target immunotherapies with different targets of action. Safety analyses revealed increased risks of any adverse events (RR=1.05; 95%CI: 1.02-1.09), grade≥3 AEs (RR=1.63; 95% CI: 1.37-1.94), serious AEs (RR=1.49; 95%CI:1.31-1.69) and AEs leading to treatment discontinuation (RR=2.49; 95% CI: 1.72-3.62) with dual-target immunotherapies.

**Conclusion:**

Our findings, based on phase III RCTs, are limited by substantial heterogeneity among included studies. Dual-target immunotherapies demonstrate superior PFS and ORR in NSCLC but are associated with increased toxicity, particularly with EGFR/MET-targeted agents. While offering a promising therapeutic advance, safety optimization and biomarker-driven patient selection are critical for clinical translation. Further trials are needed to validate long-term survival benefits and refine risk-benefit profiles.

**Systematic review registration:**

https://www.crd.york.ac.uk/prospero/, identifier CRD420251005168.

## Introduction

Lung cancer remains the leading cause of cancer-related mortality worldwide, with non-small cell lung cancer (NSCLC) accounting for approximately 85% of all cases (1, 2). Despite advancements in targeted therapies and immune checkpoint inhibitors (ICIs), the prognosis for advanced or metastatic NSCLC remains poor, with a 5-year survival rate below 20% (3). While therapies targeting EGFR, ALK, and PD-1/PD-L1 pathways have improved outcomes in specific patient subsets, intrinsic or acquired resistance, limited biomarker-driven eligibility, and heterogeneous treatment responses persist as major clinical challenges (4). These unmet needs underscore the urgency to develop novel therapeutic strategies with enhanced efficacy and tolerable safety profiles.

Dual-target immunotherapies represented by bispecific antibodies (BsAbs) is a promising class of immunotherapies designed to engage two distinct molecular targets simultaneously. By bridging tumor-associated antigens (TAAs) with immune effector cells or dual-blocking immune checkpoints, dual-target immunotherapies aim to amplify antitumor activity while overcoming resistance mechanisms observed with monoclonal antibodies (5). For instance, amivantamab, a BsAb targeting EGFR and MET, has demonstrated clinical activity in EGFR exon 20 insertion-mutated NSCLC, leading to its recent regulatory approval (6). Similarly, PD-1/CTLA-4-targeting BsAbs are being explored to enhance immune activation compared to monotherapy approaches (7). Despite this progress, the clinical benefits of dual-target immunotherapies in NSCLC remain inconsistent across trials, with variability in patient selection, dosing regimens, and endpoint definitions. Furthermore, safety concerns like adverse events (AEs) need systematic evaluation to optimize risk-benefit assessments.

Existing meta-analyses have primarily focused on monoclonal antibodies or small-molecule inhibitors in NSCLC, leaving the role of dual-target immunotherapies inadequately synthesized (8–11). Therefore, there is no clear conclusion whether dual-target immunotherapies can achieve an equal or superior effect compared to conventional therapies. A comprehensive evaluation of randomized controlled trials (RCTs) is critical to quantify pooled efficacy outcomes and safety profiles across diverse dual-target immunotherapies platforms. This systematic review and meta-analysis aims to consolidate evidence from RCTs to address two key questions: (1) What is the magnitude of clinical benefit offered by dual-target immunotherapies compared to standard therapies in NSCLC? (2) How do safety profiles vary among dual-target immunotherapies with conventional therapies? The findings will inform clinical decision-making, guide future trial design, and identify knowledge gaps for further investigation.

## Methods

### Search strategy

The present study strictly complied with the relevant requirements of the PRISMA guidelines and completed the PRISMA checklist (12). The study protocol was prospectively registered in the PROSPERO database (registration number: CRD420251005168) and was previously published. A systematic literature search was conducted in Pubmed, Web of Science, Scopus and Embase for studies published before March 2025 that compared dual-target immunotherapies and conventional therapies, using the following searching terms: Bispecific antibodies, BsAbs, lung cancer, NSCLC. The detailed search strategy is available in **Supplementary Material**. In addition, the references of all relevant articles were also searched to find additional literature. Only the studies in English were included.

### Inclusion criteria and exclusion criteria

Studies were selected based on the following inclusion criteria: (1) Phase III randomized controlled trials (RCTs) comparing dual-target immunotherapies with conventional therapeutic regimens in non-small cell lung cancer (NSCLC) populations; (2) Availability of essential statistical parameters for meta-analysis, including at minimum one clinically validated endpoint: progression-free survival (PFS), overall survival (OS), objective response rate (ORR), or disease control rate (DCR); (3) Peer-reviewed full-text manuscripts with extractable outcome data; (4) Publications in English with accessible methodological details.

Exclusion criteria comprised: (1) Early-phase clinical trials (phase I/II studies); (2) Non-original research including editorials, narrative reviews, preclinical investigations, case reports, and commentary articles; (3) Therapeutic interventions utilizing non-BsAb-based strategies or studies lacking comparator arms; (4) Trials with incomplete statistical reporting preventing quantitative synthesis.

### Data extraction

Two investigators independently performed study screening and data extraction in duplicate following the predefined inclusion/exclusion criteria. All pertinent data were systematically extracted using standardized forms, followed by cross-verification of the results. Any discrepancies in data interpretation were resolved through consensus discussions, with unresolved cases adjudicated by a third senior researcher. The following data were collected from each study: first author, publication year, NCT identifier, sample size, sex, age, PFS, OS, ORR, DCR, any adverse events (AEs), grade ≥3 AEs, serious AEs and AEs leading to treatment discontinuation.

### Risk of bias assessment

The methodological quality of included studies was evaluated using the Cochrane Collaboration’s Risk of Bias Tool (RoB 2.0) through RevMan 5.4 software. Two independent reviewers assessed seven domains: (1) random sequence generation (selection bias); (2) allocation concealment (selection bias); (3) blinding of participants and personnel (performance bias); (4) blinding of outcome assessment (detection bias); (5) incomplete outcome data (attrition bias); (6) selective reporting (reporting bias); (7) other potential sources of bias. Each domain was judged as “low risk”, “unclear risk”, or “high risk” (13). Discrepancies were resolved through consensus or consultation with a third investigator.

### Statistical analysis

Hazard ratios (HRs) with corresponding confidence intervals (CIs) were extracted as primary measures for overall survival (OS) and progression-free survival (PFS). Binary endpoints including AEs and DCR were quantified using risk ratio (RR) with 95% CIs. The *I^2^* statistics were utilized to evaluate the heterogeneity. *I^2^* < 25%, 25% ≤ *I^2^* ≤ 50%, and *I^2^* > 50% were regarded as low, moderate, and high heterogeneity. Given the substantial variability in methodological approaches observed across enrolled trials, a random-effects model was employed for all quantitative syntheses to account for potential between-study heterogeneity, irrespective of initial heterogeneity assessment results. To assess the robustness of outcomes with statistically significant and substantial heterogeneity (p ≤ 0.05, *I²* > 50%), ​leave-one-out sensitivity analyses​ were performed. Pooled estimates (HR for PFS; RR for dichotomous outcomes) and *I²* statistics were recalculated after sequentially excluding each included trial, maintaining original random-effects models (14, 15). Subgroup analyses stratified according to the different mechanisms of dual-target immunotherapies were performed to assess differences between different BsAbs or bifunctional fusion protein while mitigating the impact of heterogeneity. Subgroup analyses were performed only for categories with ≥2 studies. Subgroups with a single study were described qualitatively.

## Results

### Selected studies and study characteristics

**Figure 1:**
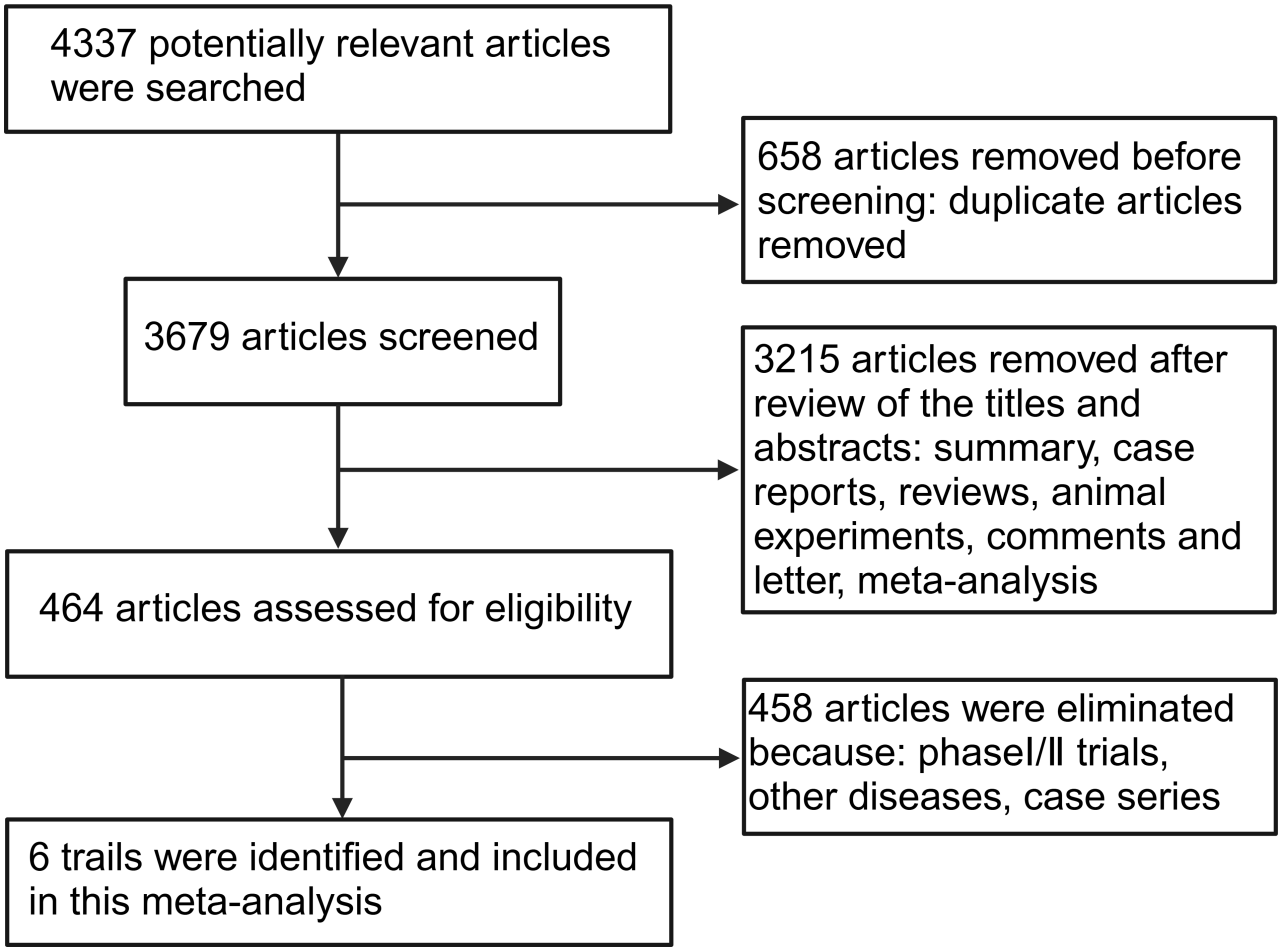
Flow diagram for study identification and selection process.

A total of 4,337 potential articles published before March 2025 were identified from databases. After removing 658 duplicates, 3,215 articles were excluded by reviewing the titles and abstracts because they were a review, summary, case report, animal experimental study, comments, or meta-analysis. 458 articles were removed because they were phase I/II trials or did not focus on NSCLC. Finally, 6 phase III RCTs met the eligibility criteria and were included in the present meta-analysis (16–21). A flow diagram of the search strategies, which includes reasons for the exclusion of articles is shown in **Figure 1**.

6 studies with a total of 3,063 patients, of which 1,224 patients were in the BsAbs group and 1,360 patients in the conventional therapy group, were involved (16–21). All the eligible studies were published between 2023 and 2025. The detailed characteristics of the included publications are summarized in **Table 1**.

**Table 1:** Main characters of included studies.

Study	Year	Therapeutic agent	Group	Cases	Age, median (range)	Sex (Male vs Female)	NCT identifier
Zhou (19)	2023	Amivantamab	Amivantamab-chemotherapy	153	61 (27-86)	68 vs 85	NCT04538664
			chemotherapy	155	62 (30-92)	62 vs 93	
Byoung (20)	2023	Bintrafusp Alfa	​Bintrafusp Alfa	152	68 (62–73)	110 vs 42	NCT03631706
			Pembrolizumab	152	68 (61–75)	116 vs 36	
Fang (16)	2024	Ivonescimab	Ivonescimab-chemotherapy	161	59.6 (32.3-74.9)	77 vs 84	NCT05184712
			placebo-chemotherapy	161	59.4 (36.2-74.2)	79 vs 82	
Passaro (17)	2024	Amivantamab	Amivantamab–lazertinib–chemotherapy	263	61 (23-83)	95 vs 168	NCT04988295
			Amivantamab–chemotherapy	131	62 (36-84)	50 vs 81	
			Chemotherapy	263	62 (31-85)	106 vs 157	
Byoung (18)	2024	Amivantamab	Amivantamab-Lazertinib	429	64 (25-88)	178 vs 251	NCT04487080
			Osimertinib	429	63 (28-88)	154 vs 275	
			​Lazertinib	216	63 (31–87)	80 vs 136	
Xiong (21)	2025	​Ivonescimab	​Ivonescimab	198	65 (37-85)	164 vs 34	NCT05499390
			Pembrolizumab	200	66 (35-83)	169 vs 31	

### Efficacy of dual-target immunotherapies

**Figure 2:**
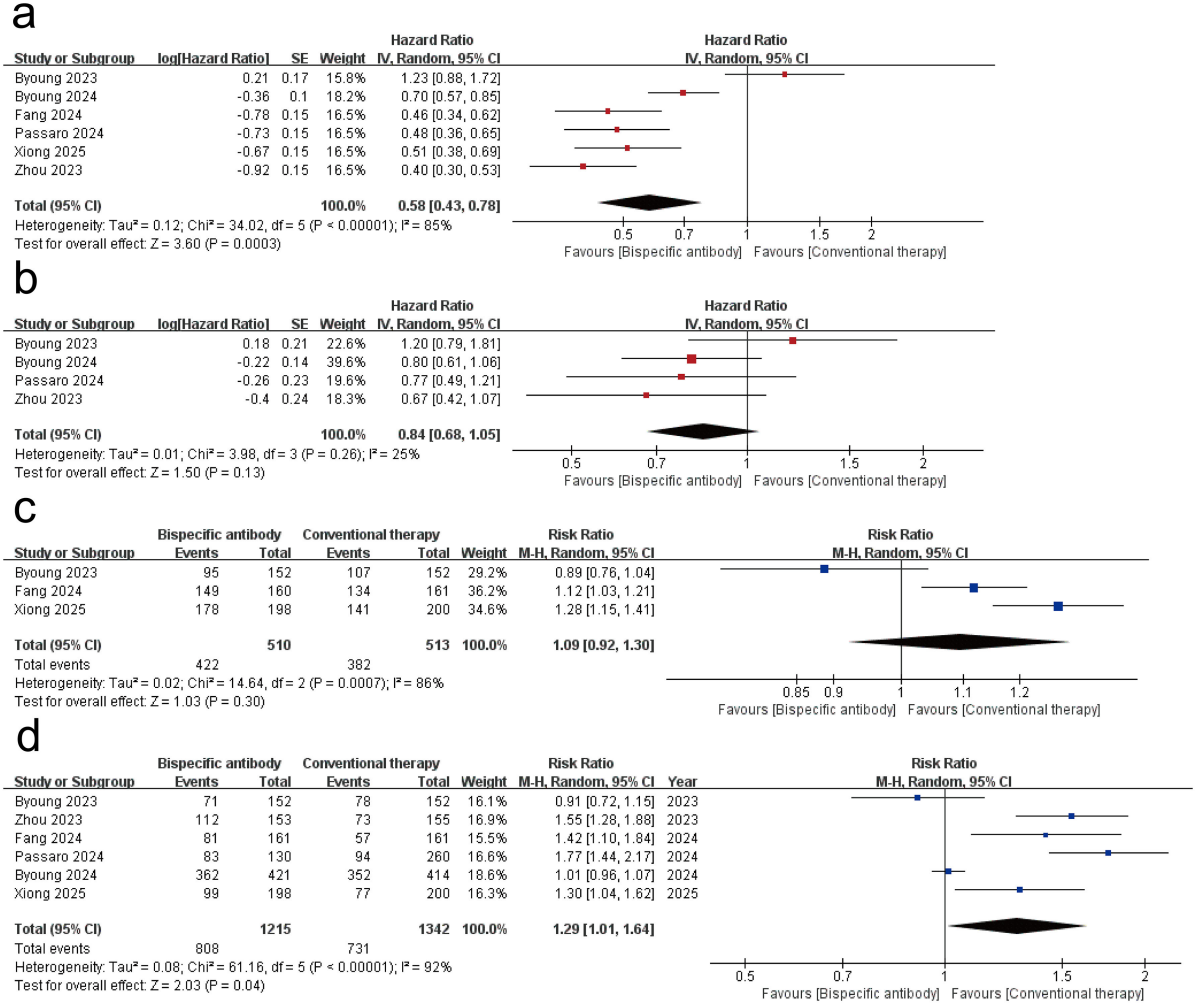
Forest plot of the meta-analysis for the efficacy of dual-target immunotherapies. **(a)** progression-free survival (PFS); **(b)** overall survival (OS); **(c)** disease control rate (DCR); **(d)** objective response rate (ORR).

All of the 6 studies reported the PFS and ORR as the main outcomes of tumor immunotherapy. 3 of the studies reported DCR (16, 20, 21), and 4 studies reported OS (17–20). **Figure 2** shows the results of the meta-analysis for the efficacy of dual-target immunotherapies. The pooled analysis revealed a statistically significant improvement in PFS with bispecific antibody therapy compared to conventional therapy, with a hazard ratio (HR) of ​0.58 (95% CI: 0.43–0.78; *P* < 0.001). Substantial heterogeneity was observed across studies (*I*² = ​85%; *P* < 0.00001), necessitating a random-effects model. The meta-analysis of four randomized trials revealed no statistically significant improvement in OS (HR = 0.84; 95% CI: 0.68–1.05; *P* = 0.13) and DCR (RR = 1.09; 95% CI: 0.92-1.30; *P* = 0.30) with bispecific antibody therapy compared to conventional treatment. A random-effects model was applied due to clinical diversity in trial designs and patient populations. The meta-analysis demonstrated a statistically significant improvement in ORR with bispecific antibodies (RR = ​1.29, 95% CI: 1.01–1.64; *P* = 0.04). Due to the high heterogeneity (*I*² = 92%; *P* = 0.04), a random-effects model was applied.

### Safety of dual-target immunotherapies

**Figure 3:**
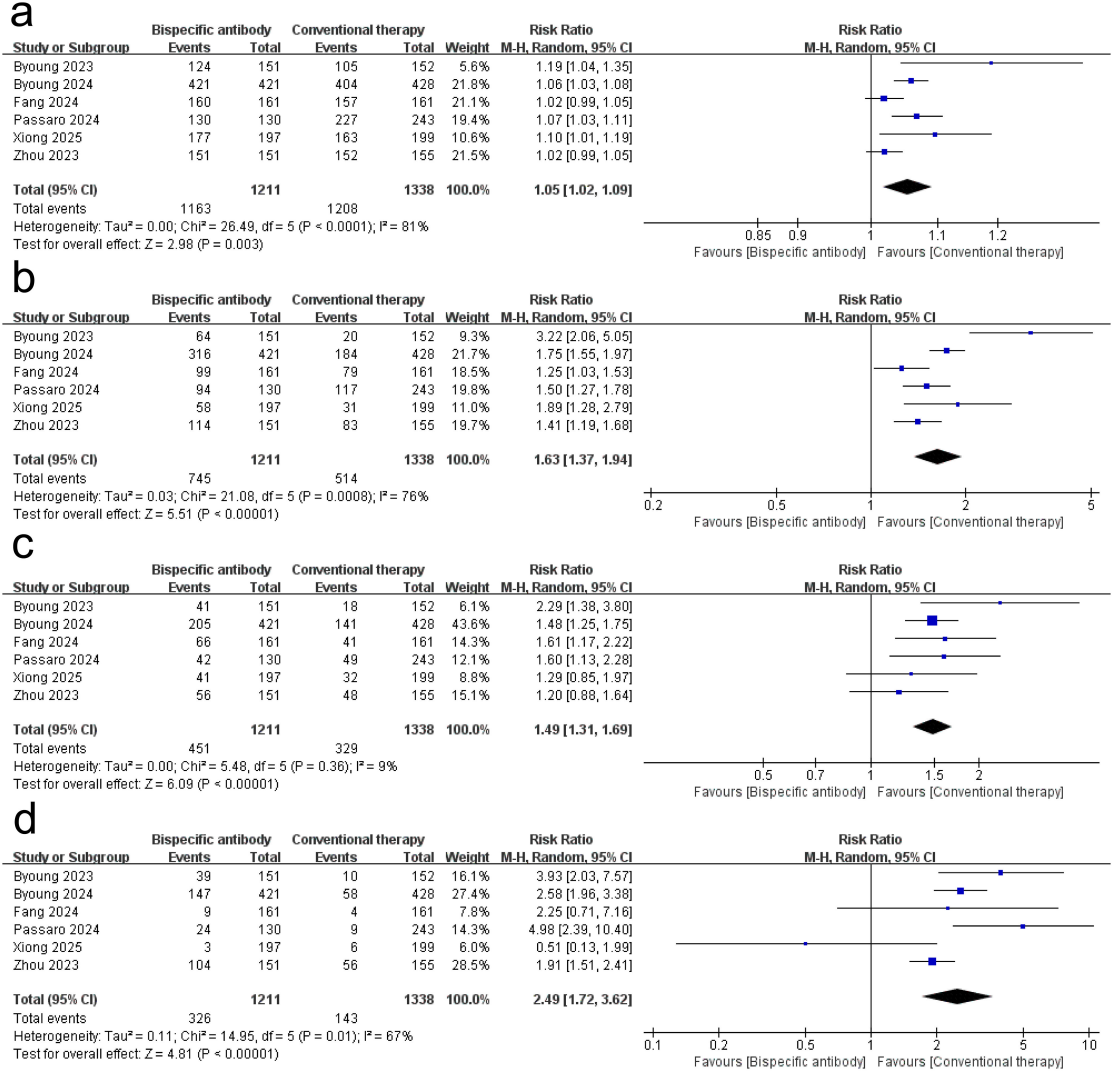
Forest plot of the meta-analysis for the safety of dual-target immunotherapies. **(a)** any AEs; **(b)** Grade ≥ 3 AEs; **(c)** serious AEs; **(d)** AEs led to treatment discontinuation.

**Figure 3** shows the results of the meta-analysis for the safety of dual-target immunotherapies. The meta-analysis of six randomized trials (dual-target immunotherapies group: *n* = 1,211; conventional therapy: *n* = 1,338) revealed a statistically significant increase in the risk of any adverse events (AEs) with dual-target immunotherapies (RR = 1.05, 95% CI: 1.02–1.09; *p* = 0.003), though with substantial heterogeneity (*I*² = 81%, *p* < 0.0001). dual-target immunotherapies ​significantly increased the risk of grade ≥ 3 AEs (RR = 1.63, 95% CI: 1.37–1.94; *p* < 0.00001; *I*² = 76%; *p* = 0.0008), serious AEs (RR = 1.49, 95% CI: 1.31–1.69; *p* < 0.00001; *I*² = 9%; *p* = 0.36), and AEs led to treatment discontinuation (RR = 2.49, 95% CI: 1.72–3.62; *p* < 0.00001; *I*² = 67%; *p* = 0.01). **Supplementary Table S6** quantifies AEs frequencies, revealing that non-chemotherapy dual-target immunotherapies regimens exhibited dermatologic event predominance, whereas BsAb-chemotherapy combinations showed hematologic burden. These findings suggest that bispecific antibody therapy is consistently associated with elevated AE risks across severity grades and clinically significant endpoints compared to conventional therapy.

### Sensitivity analysis

Sensitivity analyses (**Supplementary Tables S1**–**S5**) demonstrated consistent PFS benefit across all exclusions (HR range: 0.51-0.63, 95% CIs excluded 1), with heterogeneity decreasing from 85% to 68% when excluding Byoung 2023 (20). ORR significance was lost upon excluding Passaro 2024 (17) (RR = 1.21, 95% CI: 0.96-1.51) or Fang 2024 (16) (RR=1.26, 95% CI: 0.97-1.66), though directional consistency persisted (RR range: 1.21-1.38; *I²* > 88%). Safety signals remained robust: any AEs (RR = 1.05-1.07), grade ≥ 3 AEs (RR = 1.52-1.73, all *p* < 0.001), and treatment-discontinuing AEs (RR = 2.24-2.73) showed persistent risk elevations, with Byoung 2023 (20) exclusion reducing heterogeneity for grade ≥ 3 AEs from 76% to 62%.

### Subgroup analysis

Subgroup analyses by dual-target immunotherapies mechanism demonstrated comparable PFS benefits between PD-1/VEGF-targeted agents (HR = 0.48, 95% CI: 0.39–0.60; *I*² = 0%, 2 trials) and EGFR/MET-targeted agents (HR = 0.52, 0.37–0.74; *I*² = 82%, 3 trials), with no significant subgroup differences (χ² = 0.11, df = 1, *P* = 0.74; *I*² = 0%). For PD-L1/TGF-β-targeted agents (1 trial), the HR of PFS was 1.23 (0.88–1.72). ORRs showed a similar situation across subgroups: PD-1/VEGF agents achieved a RR of 1.39 (1.18–1.65; *I*² = 0%), while EGFR/MET agents showed an RR of 1.40 (0.89–2.18; *I*² = 96%), with no subgroup interaction (χ² = 0.00, *P* = 1.00) (**Supplementary Figures S1**, **S2**).

Analyses stratified by mechanism revealed homogeneous risks for any-grade adverse events (AEs) (PD-1/VEGF: RR = 1.05, 0.93–1.19; EGFR/MET: RR = 1.05, 1.02–1.08; subgroup *P* = 0.93; *I*² = 0%) and serious AEs (PD-1/VEGF: RR = 1.48, 1.15–1.92; EGFR/MET: RR = 1.44, 1.26–1.65; subgroup *P* = 0.83; *I*² = 0%). For grade ≥3 AEs, both subgroups exhibited elevated risks (PD-1/VEGF: RR = 1.49, 0.98–2.25; EGFR/MET: RR = 1.56, 1.37–1.79; subgroup *P* = 0.83; *I*² = 0%). EGFR/MET-targeted agents demonstrated a higher numerical risk for treatment discontinuation due to AEs (RR = 2.56, 1.73–3.80) compared to PD-1/VEGF agents (RR = 1.12, 0.26–4.82), though the subgroup difference was nonsignificant (χ² = 1.15, *P* = 0.28; *I*² = 13.2%). All analyses utilized random-effects models (**Supplementary Figures S3**–**S6**).

### Risk of bias

**Figure 4:**
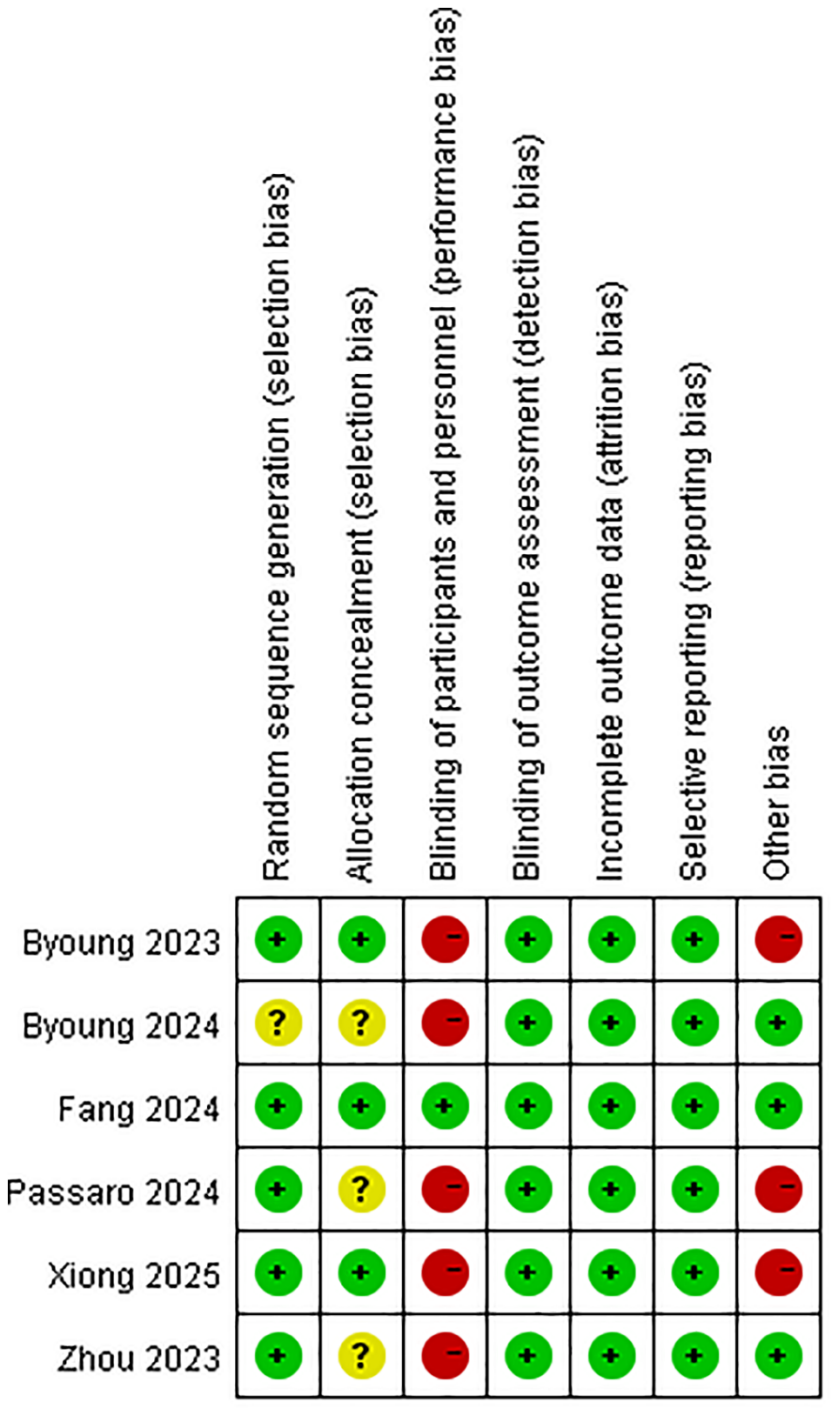
Quality assessment of the included studies according to the Cochrane Collaboration’s risk of bias tool 2 (RoB2).

The methodological quality of included randomized trials was assessed using the Cochrane Risk of Bias Tool (**Figure 4**). Four studies met ≥ 5/7 low-risk criteria (16, 19–21). Major limitations involved blinding deficiencies in 50% of trials.

## Discussion

This systematic review and meta-analysis of 6 randomized controlled trials involving 3,063 patients provides the first comprehensive evaluation of dual-target immunotherapies in advanced or metastatic NSCLC. Our findings demonstrate that dual-target immunotherapies significantly improve PFS (HR = 0.58; 95% CI: 0.43-0.78) and ORR (RR = 1.29; 95% CI: 1.01-1.64) compared to conventional therapies, though no statistically significant benefits were observed for OS (HR = 0.84; 95% CI: 0.68-1.05) or DCR (RR = 1.09; 95% CI: 0.92-1.30). These results suggest that dual-target immunotherapies confer clinically meaningful antitumor activity, particularly in delaying disease progression and enhancing tumor shrinkage, but their impact on long-term survival outcomes remains uncertain. Safety analyses revealed increased risks of any adverse events (RR = 1.05; 95% CI: 1.02-1.09) with dual-target immunotherapies and statistically significant differences in grade ≥ 3 AEs (RR = 1.63; 95% CI: 1.37-1.94) and serious AEs (RR = 1.49; 95% CI: 1.31-1.69). Moreover, treatment discontinuation rates (RR = 2.49; 95% CI: 1.72-3.62) also showed a significant difference. Collectively, these findings position dual-target immunotherapies as a dual-edged therapeutic advance in NSCLC, offering clinically meaningful antitumor activity that necessitates judicious integration into treatment algorithms through biomarker-guided patient selection and proactive toxicity mitigation strategies. A previous Meta-analysis of BsAbs for the treatment of solid tumors illustrated no significant improvement in safety or efficacy outcomes for BsAbs compared to conventional therapies and is not consistent with the results presented here (22), a discrepancy that may be the result of strong confounding factors introduced by multiple cancers. In addition, BsAbs led to an increased incidence of adverse events represented by infections when treating lymphoma (23). This is consistent with our findings, revealing that the incidence and severity of adverse events should be considered when assessing the benefit of these therapies. Previous reviews on the application of BsAbs in the treatment of NSCLC have mainly focused on the mechanism of action of BsAbs, and this narrative approach lacks a quantitative description of their clinical efficacy and safety (24–28). Furthermore, comprehensive reviews exist that delve into the synergistic potential of BsAbs when combined with chemotherapy, while also offering more thorough analyses of the future challenges confronting BsAbs development and clinical implementation (29, 30). The majority of current meta-analyses for BsAbs focus predominantly on hematological malignancies (31–35), while investigations into solid tumors, particularly NSCLC, remain comparatively scarce (22). This meta-analysis significantly advances the understanding of dual-target immunotherapies in NSCLC beyond existing reviews by ​consolidating diverse clinical datasets​ to establish a quantitative efficacy-toxicity framework, bridging mechanistic insights with clinically actionable evidence for treatment decision-making.

**Figure 5:**
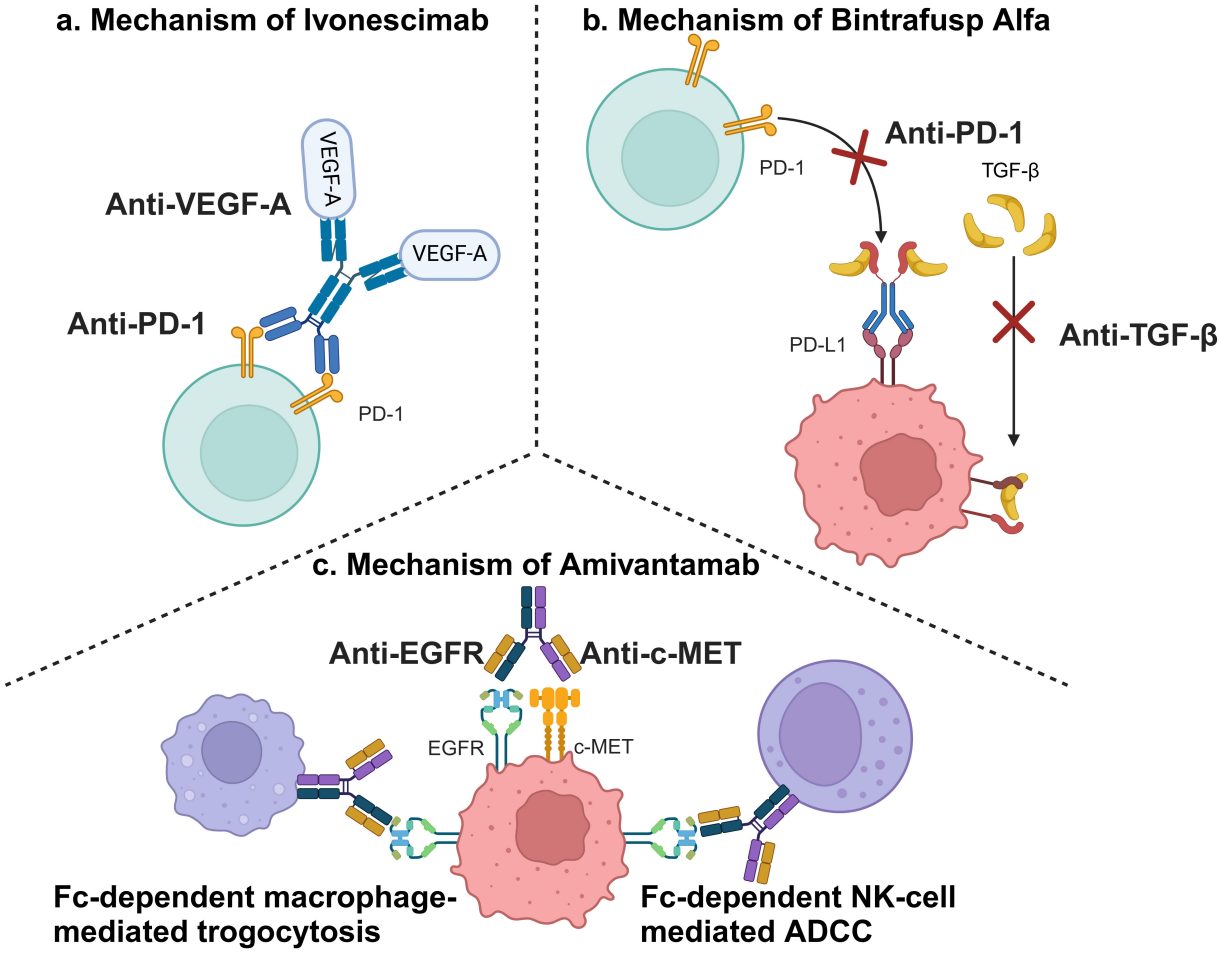
Mechanisms of involved dual-target immunotherapies. **(a)** Mechanism of Ivonescimab; **(b)** Mechanism of Bintrafusp Alfa; **(c)** Mechanism of Amivantamab (This figure was created by Biorender.).

The analysis revealed substantial heterogeneity across studies (*I²* = 85% for PFS, *P* < 0.001; *I²* = 92% for ORR, *P* < 0.001), a critical methodological challenge that complicates the interpretation of pooled efficacy outcomes. The sensitivity analyses (**Supplementary Tables S1**–**S5**) collectively affirm the ​robustness of PFS benefit​ (HR consistently < 0.63 despite high baseline heterogeneity *I²* = 85%), with Byoung 2023 (20) identified as a key contributor to variability potentially attributable to its PD-L1-enriched cohort design. ​ORR fragility manifested as loss of statistical significance when excluding Passaro 2024 (17) (RR = 1.21, 95%CI: 0.96-1.51) or Fang 2024 (16) (RR = 1.26, 95%CI: 0.97-1.66) exposing critical limitations in response assessment standardization across trials (residual *I²* > 88%). Most critically, ​immutable safety signals​ persist with treatment-discontinuing AEs maintaining RR > 2.24 in all iterations (peaking at RR = 2.73 when excluding Zhou 2023 (19)), demanding proactive toxicity management protocols irrespective of trial heterogeneity. These findings validate the random-effects model’s adequacy while underscoring biological diversity in dual-target immunotherapies mechanisms as the primary heterogeneity source, necessitating biomarker-stratified studies for future precision applications. This heterogeneity likely originates from fundamental differences in therapeutic mechanisms among evaluated dual-target immunotherapies. The three agents (two BsAbs + one bifunctional fusion protein) involved in this study can be found shown in **Table 1**. Ivonescimab simultaneously blocks the binding of PD-1 to its ligand (PD-L1), thereby alleviating PD-1/PD-L1-mediated immunosuppression, and the binding of vascular endothelial growth factor (VEGF)-A to its receptor (VEGFR2), thereby blocking tumor angiogenesis in the tumor microenvironment (36). Amivantamab is a BsAb targeting EGFR and MET, which can bind to both EGFR and c-MET sites outside of tumor cells and also kill tumor cells through mechanisms such as Fc-mediated antibody-dependent cell-mediated cytotoxicity (ADCC) effect (37). Bintrafusp Alfa is an innovative bifunctional fusion protein consisting of the extracellular structural domain of human transforming growth factor beta receptor II (TGF-βRII) fused to an IgG1 antibody that blocks PD-L1. This unique design enables it to inhibit TGF-β and PD-L1 immunosuppressive pathways, thereby enhancing anti-tumor immune responses (38). The mechanisms of action of the three dual-target immunotherapies were well illustrated in **Figure 5**.

Substantial heterogeneity arose from divergent mechanisms: Amivantamab’s efficacy was mutation-dependent, Ivonescimab relied on PD-L1 expression, and Bintrafusp Alfa’s dual pathway inhibition lacked predictive biomarkers. Variability in trial designs (e.g., combination therapies, line of treatment) and patient populations (e.g., EGFR/ALK status, cancer grading) further contributed to heterogeneity. In addition, the mode of administration is an important factor affecting the safety. A phase III study comparing subcutaneous and intravenous Amivantamab demonstrated that subcutaneous administration had a longer OS, lower risk of infusion-related reactions (IRRs), and higher end-of-treatment rates, demonstrating non-inferiority overall (39). However, our meta-analysis is fundamentally limited by the exclusive use of intravenous therapy across all included trials. This uniform delivery method restricts the generalizability of our safety and efficacy findings to subcutaneous formulations, which are emerging as a clinically advantageous alternative due to reduced IRRs and improved patient compliance (40). Although all six included trials exclusively utilized intravenous infusion for dual-target immunotherapies, which somewhat attenuated the heterogeneity, differences in the dose ranges of the therapies and the dosing cycles still contribute to the heterogeneity of the study. Critically, the exclusive intravenous administration in all trials may confound safety outcomes. Subcutaneous delivery—with slower drug release and lower peak concentrations—potentially reduces acute toxicities like cytokine release syndrome (40, 41). Conversely, intravenous infusion likely amplified the elevated AE risks observed in our pooled analysis. This implies our reported toxicity profiles may partially reflect delivery methods rather than inherent therapeutic effects. Direct comparisons of administration routes are urgently needed. A notable source of heterogeneity stems from the inclusion of ​structurally distinct agents, such as bifunctional fusion proteins (e.g., bintrafusp alfa targeting PD-L1/TGF-β) alongside canonical bispecific antibodies. Although these agents share a common mechanistic principle of dual-target engagement, differences in molecular architecture may influence pharmacokinetics, effector functions, and toxicity profiles (42). This heterogeneity is an inherent limitation of our broadened scope but reflects real-world clinical diversity in emerging immunotherapies.

We acknowledge the limitations highlighted by the RoB2 assessment and their potential impact on the interpretation of our findings (43). As noted in **Figure 4**, the primary methodological concerns arose from deficiencies in blinding (performance bias) and, to a lesser extent, potential attrition bias in some trials. The lack of blinding could amplify efficacy estimates for investigator-assessed endpoints: awareness of treatment allocation may systematically influence tumor response evaluations, potentially inflating observed PFS benefits (HR = 0.58) and ORR advantages (RR = 1.29). Concurrently, heightened AE vigilance in the dual-target immunotherapies arm may overstate safety risks (e.g., any-grade AE RR = 1.05; grade ≥ 3 AE RR = 1.63). Attrition bias warrants consideration given significantly higher dual-target immunotherapies discontinuation rates (RR = 2.49). Disproportionate dropout may dilute survival signals—as subsequent therapies could obscure true OS benefits (HR = 0.84)—and skew time-to-event analyses. While these biases preclude definitive quantification, they necessitate cautious interpretation: efficacy advantages may be overestimated, and AE magnitudes may reflect detection artifacts. Consequently, our results should be contextualized as potentially influenced by inherent trial limitations, underscoring the need for future studies to prioritize blinding strategies and rigorous attrition management.

While exploratory subgroup analyses suggested potential efficacy differences by dual-target immunotherapies mechanism, the small number of studies per subgroup (n ≤ 3) precludes definitive conclusions. Given the limited studies per subgroup, these findings are hypothesis-generating and require validation in larger cohorts (44, 45).

Most of the current clinical studies on dual-target immunotherapies are in phase I or II, which have a limited role in assessing the benefits and risks of this therapy (46–52). Further high-quality randomized controlled trials of dual-target immunotherapies in solid tumors are strongly recommended to better evaluate the clinical potential of this therapy.

## Conclusion

Dual-target immunotherapies ​confer superior efficacy in delaying disease progression and tumor response compared to conventional NSCLC therapies, but their elevated toxicity risk profiles require​ biomarker-driven patient selection to optimize clinical implementation.

## Data Availability

The original contributions presented in the study are included in the article/**Supplementary Material**. Further inquiries can be directed to the corresponding author.
