# Supplementary material for: Dual-target immunotherapies in NSCLC: a systematic review and meta-analysis of randomized clinical trials
Source: Front Immunol. 2025 Sep 10;16:1605877. doi: 10.3389/fimmu.2025.1605877 (PMC12457429; doi:10.3389/fimmu.2025.1605877)
Supplement: Supplementary file 3 [file Table3.docx]

**Dual-target Immunotherapies in NSCLC: A Systematic Review and Meta-analysis of Randomized Clinical Trials**

Yike Zhang^1†^, Haozhe Wang^1†^, Xinyue Yang^1†^, Changhai Lei^1^

1 School of Basic Medical Sciences, Naval Medical University, No 800. Xiangyin Road, Yangpu, Shanghai, 200433, China. [lei@smmu.edu.cn](mailto:lei@smmu.edu.cn)

**1. Supplementary Methods**

**1.1 Search Strategy**

**1.1.1 Pubmed**

Search:(((((Lung Neoplasms) OR (Lung Cancer)) OR (NSCLC)) OR(SCLC))OR (pulmonary carcinoma)) AND(((Bispecific Antibodies) OR(BsAb))OR (dual-target antibody)) Sort by: Most Recent

**1.1.2 Web of science**

#1 ((TS=(Bispecific antibodies)) OR TS=(BsAb)) OR TS=(dual-target antibody) andPreprint Citation Index (Exclude -Database)

#2 ((((TS=(Lung Neoplasms)) OR TS=(Lung Cancer)) OR TS=(NSCLC)) OR TS=(SCLC)) ORTS=(pulmonary carcinoma) and Preprint Citation Index (Exclude - Database)

#3 #2 AND #1 and Preprint Citation Index (Exclude -Database)

**1.1.3 Embase**

#1 bispecific AND ('antibodies'/exp OR antibodies)

#2 bsab

#3 'dual-target antibody' OR ('dual target' AND ('antibody'/exp OR antibody))

#4 #1 OR #2 OR #3

#5 'lung neoplasms'/exp OR 'lung neoplasms' OR (('lung'/exp OR lung) AND ('neoplasms'/exp OR neoplasms))

#6 'lung cancer'/exp OR 'lung cancer' OR (('lung'/exp OR lung)AND ('cancer'/exp OR cancer))

#7 nsclc

#8 sclc

#9 'pulmonary carcinoma'/exp OR 'pulmonary carcinoma' OR (pulmonary AND ('carcinoma'/exp OR carcinoma))

#10 #5 OR #6 OR #7 OR #8 OR #9

#11 #4 AND #10

**1.1.4 Scopus**

( ( TITLE-ABS-KEY ( bsab ) OR TITLE-ABS-KEY ( bispecific AND antibodies ) OR TITLE-ABS-KEY ( dual-target AND antibody ) ) ) AND ( ( TITLE-ABS-KEY ( lung AND cancer ) OR TITLE-ABS-KEY ( lung AND neoplasms ) OR TITLE-ABS-KEY ( nsclc ) OR TITLE-ABS-KEY ( sclc ) OR TITLE-ABS-KEY ( pulmonary AND carcinoma ) ) ) AND ( EXCLUDE ( DOCTYPE , "re" ) OR LIMIT-TO ( DOCTYPE , "ar" ) )

**2. Supplementary Results**

**2.1 Sensitivity analysis**

**Table S1. Leave-One-Out Sensitivity Analysis of Pooled HR and Heterogeneity (I²) for PFS**

| **Excluded Study** | **Pooled HR (95% CI)** | ***I²* (%)** |
| --- | --- | --- |
| None (Baseline) | 0.58 [0.43, 0.78] | 85 |
| Byoung 2023 | 0.51 [0.41, 0.63] | 68 |
| Byoung 2024 | 0.56 [0.39, 0.80] | 86 |
| Fang 2024 | 0.61 [0.43, 0.86] | 87 |
| Passaro 2024 | 0.60 [0.42, 0.85] | 88 |
| Xiong 2025 | 0.60 [0.42, 0.85] | 88 |
| Zhou 2023 | 0.63 [0.46, 0.86] | 85 |

**Table S2. Leave-One-Out Sensitivity Analysis of Pooled RR and Heterogeneity (I²) for ORR**

| **Excluded Study** | **Pooled RR (95% CI)** | **I² (%)** |
| --- | --- | --- |
| None | 1.29 [1.01, 1.64] | 92 |
| Byoung 2023 | 1.38 [1.02, 1.86] | 94 |
| Zhou 2023 | 1.24 [0.96, 1.60] | 91 |
| Fang 2024 | 1.26 [0.97, 1.66] | 93 |
| Passaro 2024 | 1.21 [0.96, 1.51] | 88 |
| Byoung 2024 | 1.36 [1.09, 1.70] | 80 |
| Xiong 2025 | 1.29 [0.97, 1.71] | 93 |

**Table S3. Leave-One-Out Sensitivity Analysis of Pooled RR and Heterogeneity (I²) for Any Adverse Events (AEs)**

| **Excluded Study** | **Pooled RR (95% CI)** | **I² (%)** |
| --- | --- | --- |
| None | 1.05 [1.02, 1.09] | 81 |
| Byoung 2023 | 1.05 [1.02, 1.07] | 72 |
| Byoung 2024 | 1.06 [1.01, 1.12] | 87 |
| Fang 2024 | 1.07 [1.02, 1.11] | 81 |
| Passaro 2024 | 1.05 [1.01, 1.10] | 85 |
| Xiong 2025 | 1.05 [1.01, 1.09] | 81 |
| Zhou 2023 | 1.06 [1.02, 1.11] | 78 |

**Table S4. Leave-One-Out Sensitivity Analysis of Pooled RR and Heterogeneity (I²) for Grade ≥ 3 Adverse Events**

| **Excluded Study** | **Pooled RR (95% CI)** | **I² (%)** |
| --- | --- | --- |
| None | 1.63 [1.37, 1.94] | 76 |
| Byoung 2023 | 1.52 [1.33, 1.74] | 62 |
| Byoung 2024 | 1.62 [1.30, 2.03] | 77 |
| Fang 2024 | 1.73 [1.43, 2.08] | 74 |
| Passaro 2024 | 1.69 [1.35, 2.11] | 81 |
| Xiong 2025 | 1.61 [1.33, 1.94] | 80 |
| Zhou 2023 | 1.71 [1.38, 2.11] | 79 |

**Table S5. Leave-One-Out Sensitivity Analysis of Pooled RR and Heterogeneity (I²) for Adverse Events Leading to Treatment Discontinuation**

| **Excluded Study** | **Pooled RR (95% CI)** | **I² (%)** |
| --- | --- | --- |
| None | 2.49 [1.72, 3.62] | 67 |
| Byoung 2023 | 2.29 [1.54, 3.41] | 67 |
| Byoung 2024 | 2.42 [1.34, 4.34] | 71 |
| Fang 2024 | 2.52 [1.67, 3.79] | 73 |
| Passaro 2024 | 2.24 [1.57, 3.21] | 61 |
| Xiong 2025 | 2.70 [1.93, 3.78] | 62 |
| Zhou 2023 | 2.73 [1.65, 4.50] | 59 |

**2.2 Subgroup Analysis**

**
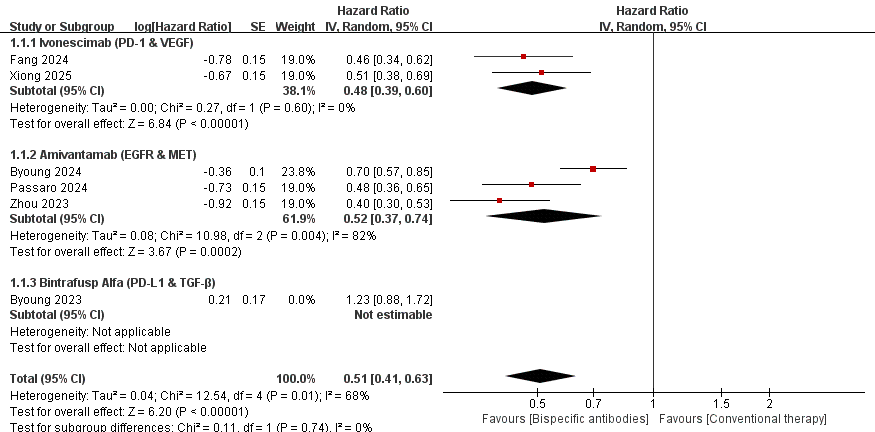
**

Figure S1. Forest plot of progression-free survival (PFS) subgroup analysis by dual-target immunotherapies mechanism


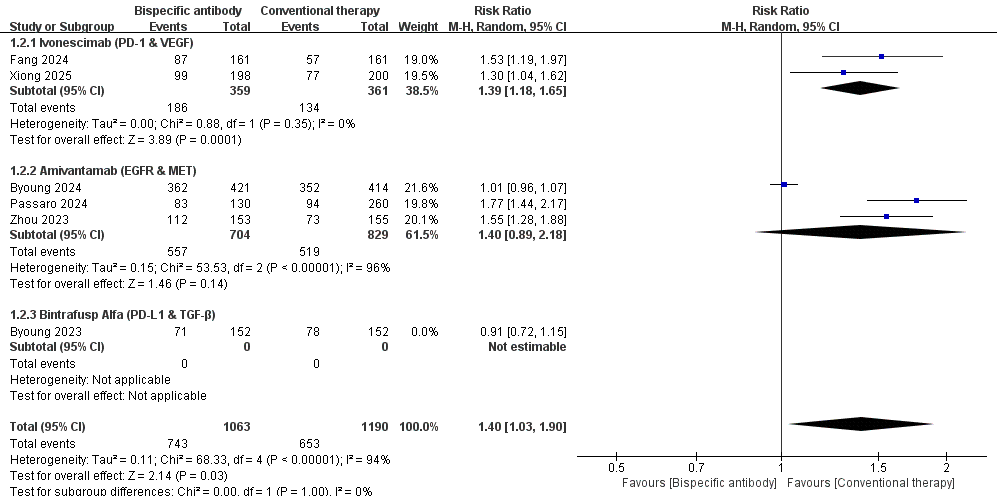


Figure S2. Forest plot of objective response rate (ORR) subgroup analysis by dual-target immunotherapies mechanism


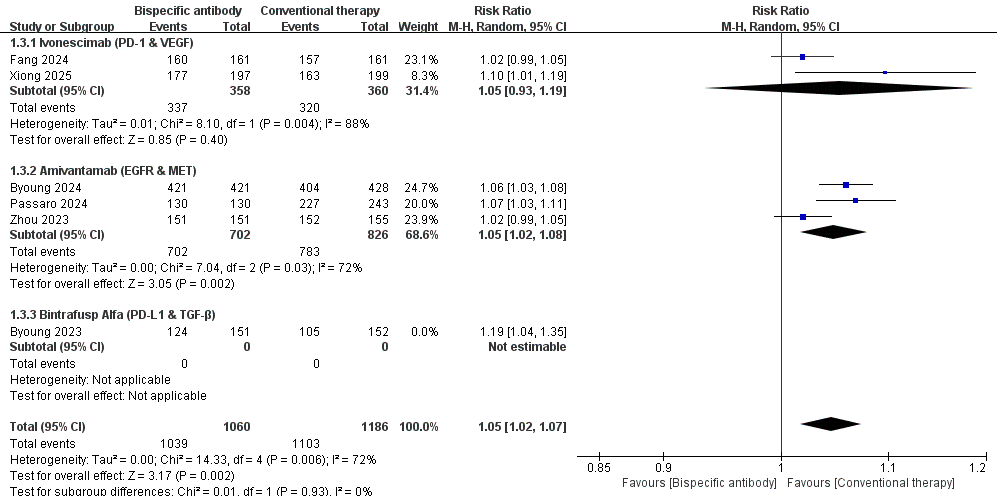


Figure S3. Forest plot of any adverse events (AEs) subgroup analysis by dual-target immunotherapies mechanism


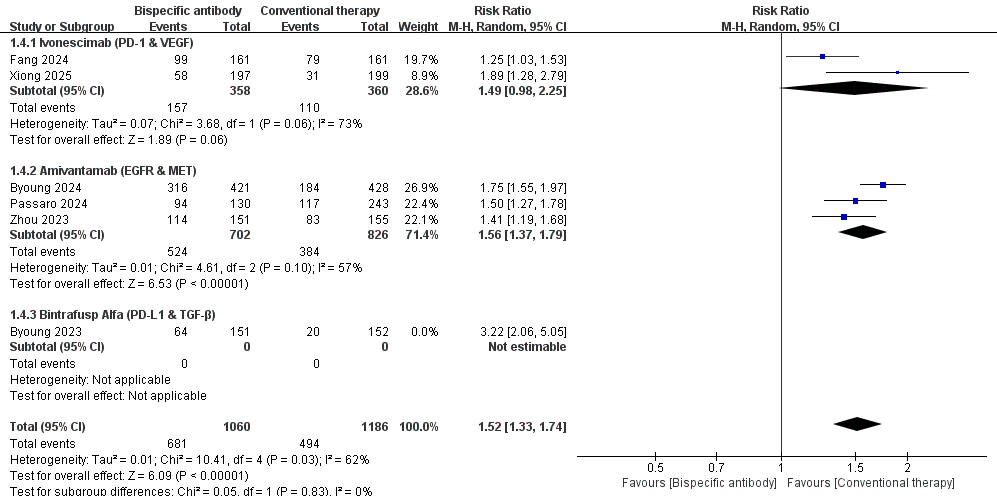


Figure S4. Forest plot of grade≥3 adverse events subgroup analysis by dual-target immunotherapies mechanism


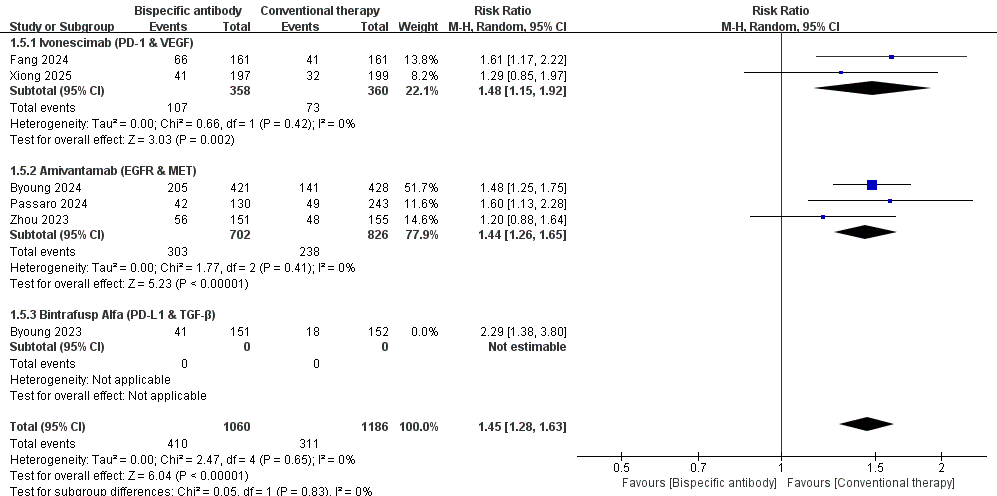


Figure S5. Forest plot of serious adverse events subgroup analysis by dual-target immunotherapies mechanism


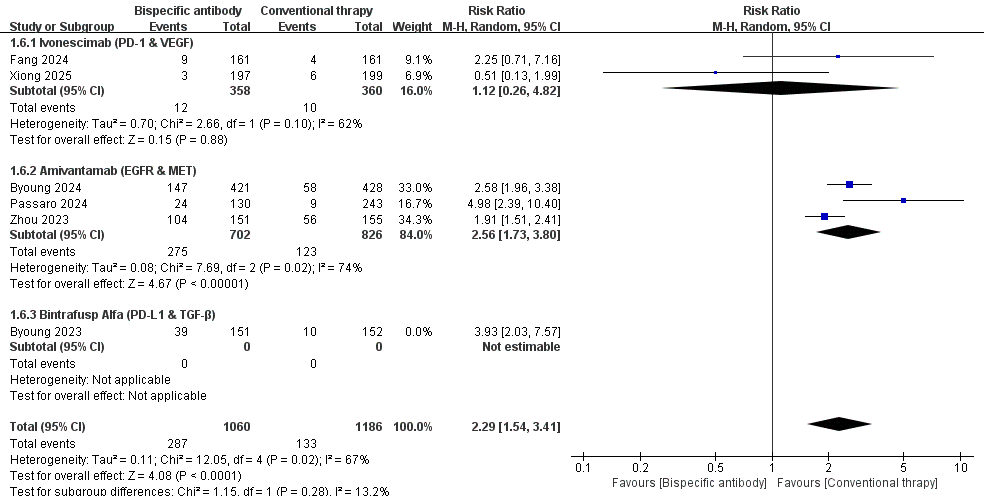


Figure S6. Forest plot of treatment discontinuation due to adverse events subgroup analysis by dual-target immunotherapies mechanism

**Table S6. Number of Infections, Skin and Hematologic Adverse Events in Six Randomized Controlled Trials**

| **Study** | **Year** | **Therapeutic Agent** | **Group** | **Cases** | **Infections** | **Dermatologic** | **Hematologic** |
| --- | --- | --- | --- | --- | --- | --- | --- |
| Zhou | 2023 | Amivantamab | Amivantamab-chemotherapy | 151 | 36 | 251 | 277 |
|  |  |  | chemotherapy | 155 | 21 | 26 | 251 |
| Byoung | 2023 | Bintrafusp Alfa | ​Bintrafusp Alfa | 151 | 0 | 161 | 16 |
|  |  |  | Pembrolizumab | 152 | 5 | 69 | 4 |
| Fang | 2024 | Ivonescimab | Ivonescimab-chemotherapy | 161 | 49 | 17 | 406 |
|  |  |  | placebo-chemotherapy | 161 | 33 | 8 | 398 |
| Passaro | 2024 | Amivantamab | Amivantamab–lazertinib–chemotherapy | 263 | 44 | 512 | 586 |
|  |  |  | Amivantamab–chemotherapy | 130 | 27 | 207 | 219 |
|  |  |  | Chemotherapy | 243 | 25 | 59 | 338 |
| Byoung | 2024 | Amivantamab | Amivantamab-Lazertinib | 421 | 111 | 958 | 188 |
|  |  |  | Osimertinib | 428 | 103 | 530 | 241 |
|  |  |  | ​Lazertinib | 216 | NA | NA | NA |
| Xiong | 2025 | ​Ivonescimab | ​Ivonescimab | 197 | NA | 18 | 26 |
|  |  |  | Pembrolizumab | 199 | NA | 34 | 29 |

Infections: Mainly includes pneumonia, COVID-19 and other adverse reactions clearly caused by pathogenic infections; Dermatologic: rash, paronychia, pruritus, dry skin, dermatitis acneiform, stomatitis and other adverse events associated with dermatologic conditions; Hematologic: anemia, leukopenia, thrombocytopenia, neutropenia and other adverse events associated with hematologic conditions.
